# A high energy flexible symmetric supercapacitor fabricated using N-doped activated carbon derived from palm flowers†

**DOI:** 10.1039/d1na00261a

**Published:** 2021-08-10

**Authors:** Malaya K. Sahoo, G. Ranga Rao

**Affiliations:** Department of Chemistry and DST-Solar Energy Harnessing Centre (DSEHC), Indian Institute of Technology Madras Chennai 600036 India grrao@iitm.ac.in +91 44 2257 4202 +91 44 2257 4226

## Abstract

Nitrogen doped activated carbons of high surface area are synthesized using palm flower biomaterial by KOH activation followed by pyrolysis. The concentration of the activating agent KOH and carbonization temperature are found to be crucial to obtain high surface area activated carbon. The optimal concentration of KOH and carbonization temperature for the synthesis of activated carbon, respectively, are 2 M and 800 °C in the flow of nitrogen gas. The optimized conditions have been employed to further prepare nitrogen doped activated carbon (NAC) by varying the weight ratio of palm flowers to melamine. All activated carbons are characterized by powder XRD, BET analysis, RAMAN spectroscopy, HR-SEM analysis, HR-TEM analysis and FT-IR analysis. With 2 wt% nitrogen doping, the BET surface area and pore diameter of the NAC-2 sample are 1054 m^2^ g^−1^ and 1.9 nm, respectively. The electrochemical charge storage performance of the nitrogen doped activated carbons has been evaluated in an aqueous acidic electrolyte medium. The results indicate that among the nitrogen doped activated carbons, the NAC-2 sample exhibits the highest electrochemical capacitance of 296 F g^−1^ at 0.5 A g^−1^. The performance of the NAC-2 electrode is further tested in aqueous, ionic liquid and solid polymer electrolytes by assembling a symmetric capacitor for real time application. By employing an ionic liquid as the electrolyte, the device delivers an energy density of 8.6 Wh kg^−1^ and a power density of 38.9 W kg^−1^ in the voltage window of 1.5 V and at an operating current density of 0.1 A g^−1^. Interestingly, the NAC-2 electrode shows good cycling performance in the ionic liquid electrolyte (up to 50k cycles). Furthermore, the symmetric device in 0.1 M H_2_SO_4_/PVA solid state electrolyte shows excellent electrochemical stability under various bending angles, demonstrating its potential in flexible electronic devices.

## Abstract 2

Nitrogen doped activated carbons of high surface area are synthesized using palm flower biomaterial by KOH activation followed by pyrolysis.

## Graphical abstract

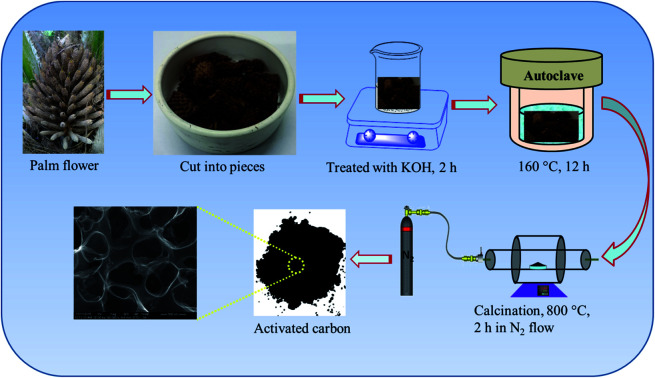

## 1. Introduction

An increasing demand for energy in today's life makes us look for sufficient and sustainable production of energy from renewable sources. But these renewable sources are dependent on days' time and weather conditions. Due to these fluctuations in renewable energy sources, development of energy-storage devices with high power densities is required for storing the available intermittent energy and supplying it on demand. Among various energy storage devices, the supercapacitor is a potential candidate, which can have power density as high as 10 kW kg^−1^, fast charge–discharge processes (within seconds) and longer life cycle (∼100 000).^1,2^ Depending on the charge storage mechanisms, supercapacitors are categorized into two types: electrochemical double layer capacitor (EDLC), which stores energy *via* surface electrostatic ion adsorption at the electrode/electrolyte interface and the second one is the pseudocapacitor, which stores energy through the faradaic process *i.e.* electron transfer between the electrolyte ion and electrode surface.^1,3,4^ It is well known that metal oxides/sulfides and conductive polymers as pseudocapacitors and carbon based materials, such as activated carbons, graphene, carbon nanotubes, carbon nanofibers, templated porous carbons, carbon-derived carbons, carbon onions, carbon hydrogels, carbon dots, amorphous carbons and carbon foams are most widely used in EDLCs.^4–10^ Currently carbon-based materials are being employed in many energy-related applications because of their abundance, chemical and thermal stability and processability.^4^ EDLCs are always an eminent choice for commercial devices owing to their simple construction, low weight, high power density and long cycle life compared to pseudocapacitors. In order to improve the capacitance value of electrode materials in EDLCs, proper control over the specific surface area and pore size distribution is essential, which can enhance the power density as well as energy storage capacity. There are several approaches in the literature aimed at raising the surface area of the electrode materials. These include chemical and physical activation, functional group modification and templating methods.^11–13^ In addition, the surface modification of carbon materials by hetero atom substitution will greatly affect the physical and chemical properties of the resulting materials. Doping a hetero atom ‘X’ (X = B, N, O, S, and F) in the carbon matrix can introduce a C–X polar bond and improve the wettability of the carbon surface.^3,14–18^ The improvement of wettability in turn enhances the conductivity of carbon materials and also the contact between the electrode and electrolyte. As a result, ions can easily diffuse from the electrolyte to electrode surface and *vice versa*.^11^ Doping a hetero atom in carbon also introduces the pseudocapacitance performance of the resulting material and the overall specific capacitance value rises by orders of magnitude.^19,20^ But excess doping with hetero atoms can collapse the pore structure of carbon, and contribute to the reduction in specific capacitance.^19^ Therefore, proper control on hetero atom doping is necessary to improve the supercapacitive performance.

In comparison to all carbon based electrodes, activated carbon has been the most admirable electrode material used for EDLCs, because of its key properties such as high surface area and micro-to-nano-porosity, which is essential for EDLCs. A number of methods have been explored for the synthesis of activated carbon *viz* carbonization, hydrothermal, pyrolysis and activation processes.^21^ Among all, activation methods are very simple and less time consuming. Generally, activation methods involve carbonization followed by chemical/physical activation depending on whether the activating agents are used or not. The morphology, surface area and pore size/structure of the activated carbon can be tailor-made by controlling the synthesis parameters such as temperature, time, pressure and an activating agent. The use of organic wastes and biomass for manufacturing activated carbons is an efficient way and eco-friendly in nature. Attempts have been made to prepare differently activated carbons from bio-sources, such as dead leaves, bamboos, coconut shells, banana fibers, corn grains, sugarcane bagasse, cotton, human hair and catkins and their charge storage performance has also been studied.^5,15,22–33^ In some countries like India, after farming, farmers burn the waste biomass, which causes huge air pollution by releasing CO and CO_2_ to the atmosphere. Instead of burning the bio-waste in local areas, these straw materials could be converted into carbon by burning under a closed inert atmosphere. Again the physical properties/activity of these bio-derived activated carbons can be enhanced by suitably engineering the synthesis process, which has been the research endeavor. There have been previous efforts aimed at synthesizing porous activated carbons through chemical activation using activating agents such as NaOH, KOH, CaO, Na_2_CO_3_, K_2_CO_3_, *etc.* which are known as porogens.^13^ It is observed that the synthesis of nanoporous carbon materials from bio-waste is an efficient way compared to complex methods of porous carbon preparation. Recently, Liu *et al.* reported nitrogen-doped hierarchical porous carbon from wheat straw with a specific surface area of 892 m^2^ g^−1^ by CaCl_2_ activation, which possessed a capacitance of 275 F g^−1^ in 6 M KOH solution.^34^ Similarly, Li *et al.* reported nitrogen-doped hierarchical porous carbon from bean curd by CH_3_COOK activation, showing a large surface area and possessed a capacitance of 284 F g^−1^ at a current density of 0.1 A g^−1^ in 6 M KOH solution.^35^ In view of the above, bio-waste derived activated carbon may be an alternative approach to fulfill the renewable energy demand in the future.

The specific energy of a supercapacitor is directly proportional to the capacitance of the cell and its operating potential window. Enhancing both cell capacitance and potential window of a cell without sacrificing power density is desirable to develop alternative energy storage devices to lithium ion batteries and fuel cells. Generally, the potential window of a device essentially depends on the nature of electrolytes used. In practice inexpensive aqueous electrolytes are employed because of the ease of handling them in the laboratory. However, aqueous electrolytes have narrow potential windows due to water decomposition at 1.23 V and are not preferred for commercial applications. Organic and ionic liquid electrolytes have large potential windows (∼3 to 4 V) compared to aqueous electrolytes. Organic electrolytes are highly flammable and thermally unstable while ionic liquids are comparatively greener and decompose at high temperatures. Hence they both are preferable to achieve high energy density in charge storage devices.^36–38^ Currently, efforts are on to develop foldable, flexible and wearable energy storage devices to meet the demand of various applications.^39–43^ It is imperative to produce flexible solid state energy storage devices using biomass-derived activated carbon which is cheaper and much easier to process.

Here, we demonstrate the fabrication of flexible symmetric capacitors using nitrogen doped activated carbon (NAC) derived from palm flower residue. The wt% of nitrogen is controlled by varying the wt% of palm flowers and melamine. The charge storage performance of NAC from palm flowers has been examined in aqueous, ionic liquid, and solid-state electrolytes. High surface area carbon materials have been extracted from palm flower residues by optimizing the concentration of KOH solution as an activating agent and temperature for the carbonization process.

## 2. Experimental section

### 2.1 Materials

Melamine and potassium hydroxide were purchased from Merck. All chemicals were of analytic grade and used without further purification. Ultrapure deionized water from a Merck Millipore water system (18.2 MΩ cm) was used in the experiments.

### 2.2 Preparation of nitrogen doped activated carbon

In a typical synthesis process, palm flowers were cut into small pieces and washed with deionized water followed by drying in an oven at 80 °C overnight. After thorough drying, stoichiometric weight ratios of palm flower/melamine were dispersed in 200 ml of 2 M KOH solution in a beaker. The contents of the beaker were stirred for 2 h and the resulting suspensions were sealed in a Teflon-lined autoclave and transferred into an oven at 160 °C. After 12 h, the autoclave was cooled to room temperature and the products were collected by centrifugation, washed with deionized water several times, and dried overnight at 60 °C. The hydrothermally activated samples were carbonized at 800 °C for 2 h under N_2_ flow with a heating rate of 5 °C min^−1^. The resulting carbon samples were soaked in 2 M HCl for 6 h, washed several times by centrifugation using deionized water and finally dried at 60 °C overnight. These activated carbons are designated as NAC-*X* (nitrogen doped activated carbon), where *X* represents the wt% of nitrogen in the activated carbon. The details of the nitrogen content present in the activated carbon samples and weight ratio of palm flower/melamine are given in Table 1.

**Table 1:** Textural properties and elemental analysis of NAC samples

Sample	Palm flower/melamine weight ratio	Elemental analysis			% weight loss (390 °C to 630 °C)	BET surface area (m^2^ g^−1^)	Pore diameter (nm)
		N (%)	C (%)	O (%)			
NAC-0	1 : 0	0	62	34	38	796	3.9
NAC-2	2 : 1	2	71	24	58	1054	1.9
NAC-4	1 : 1	4	70	25	44	793	4.0

### 2.3 Characterization

The powder XRD measurements were acquired using a Bruker AXS D8 Advance diffractometer, using Cu Kα (*λ* = 0.15408 nm) radiation. FT-IR analysis was performed using a Bruker Optic (TENSOR-27) instrument. The samples mixed with KBr were employed to record IR spectra. Raman spectra were recorded using a confocal Raman spectroscope (WiTec GmbH CRM 200). Elemental analysis was performed with a PerkinElmer 2400 Series. Conductivity of samples in the pellet form was measured using a Keysight U3606B multimeter. Thermogravimetry analyses of all activated carbons were performed on a TA TGA Q500 V20.10 Build 36 instrument under 20 ml min^−1^ air flow with a linear heating rate of 20 °C min^−1^. Nitrogen adsorption–desorption measurements were carried out with an automatic Micromeritics ASAP 2020 analyzer, using the Brunauer–Emmett–Teller (BET) gas adsorption method. The samples were degassed at 200 °C for 12 h in a vacuum before physisorption measurements at 77 K. The porosity distributions in the samples were generated from desorption branches of the isotherms, using the Barrett–Joyner–Halenda (BJH) method. The surface morphology of nitrogen doped activated carbon samples was examined by high-resolution scanning electron microscopy (HR-SEM; FEI Quanta FEG 200). The High Resolution Transmission Electron Microscopy (HRTEM) measurements were obtained with a JOEL JEM 3010 microscope which was operated at an accelerating voltage of 300 kV. The sample was sonicated in ethanol for 2 to 5 min and deposited on a copper grid for HRTEM analysis. Relative humidity of the H_2_SO_4_/PVA polymer membrane was measured with an HTC digital hygrometer.

### 2.4 Electrochemical measurements

The electrochemical behavior of nitrogen doped activated carbon samples (NAC-0, NAC-2 and NAC-4) from palm flowers was studied with both three- and two-electrode configurations in an aqueous 0.1 M H_2_SO_4_ electrolyte using a CHI 7081C electrochemical workstation. For the three-electrode system, a 5 mm dia glassy carbon electrode coated with NAC-*X* samples was used as the working electrode, and platinum foil and Ag/AgCl saturated with KCl were used as the counter and reference electrodes, respectively. Ideally the Hg/Hg_2_SO_4_ electrode is to be used as a reference electrode in aqueous H_2_SO_4_ electrolyte to avoid liquid junction potential. However, the use of the Ag/AgCl saturated with KCl reference electrode is also accepted in acidic electrolytes.^15,18,25,28,31^ The working electrode was prepared by dispersing 10 mg of active material in 600 μL H_2_O, 300 μL isopropanol and 100 μL of 5% Nafion solution followed by sonication for 6 h. A 10 μL aliquot of electrode materials was spread over the clean glassy carbon electrode (polished with 0.05 μm γ-alumina micropolish). The solvent of the aliquot was evaporated at 50 °C for 8 h and dipped in the aqueous electrolyte for 1 h before starting the experiment. The electrochemical properties of the electrode materials were studied by cyclic voltammetry (CV), galvanostatic charge discharge (GCD) and electrochemical impedance spectroscopy (EIS) analysis. The specific capacitance values of all electrodes were calculated by1
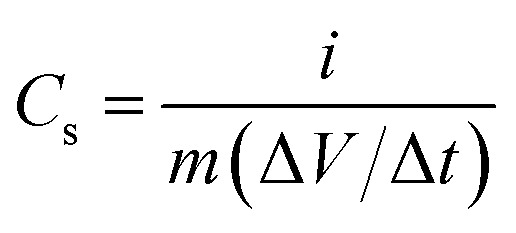
where *C*_s_ is the specific capacitance (F g^−1^), *i* is the applied current (A), *m* is the active mass of the sample, Δ*t* is the corresponding discharge time (s) and Δ*V* is the operating potential window. For the electrochemical test in the two-electrode system, the working electrode slurry was prepared by mixing 95 : 5 weight ratio of NAC-*X* samples and polyvinylidene difluoride (PVdF) binder dissolved in 1-methyl-2-pyrrolidinone. The obtained slurry was coated on carbon cloth and dried in an oven at 60 °C for 8 h. The electrode contained approximately 2 mg of active material. The two electrodes having a similar mass loading of active material were separated by a filter paper, which was fully soaked with 0.1 M H_2_SO_4_ electrolyte. The open circuit potential values of all nitrogen doped activated carbons are around 0.58 V. Nitrogen doping can enhance the electrochemical properties of carbons but does not affect the open circuit potential significantly.

In order to study the potential applications of the as synthesized nitrogen doped activated carbon in supercapacitors, a device is fabricated in a nonaqueous electrolyte (ionic liquid electrolyte), and gel electrolyte (0.1 M H_2_SO_4_/PVA). In both the cases, carbon cloth was taken as the current collector. A filter paper soaked with the ionic liquid was used as the separator in the former case, while a solid polymer electrolyte (0.1 M H_2_SO_4_/PVA) was used as the separator for flexible supercapacitor studies. H_2_SO_4_/PVA polymer electrolyte was prepared by dissolving 1 g of PVA in 20 ml H_2_O with continuous stirring at 80 °C. Then 20 ml of 0.1 M H_2_SO_4_ was added to the above solution and stirred continuously to get a homogeneous viscous solution. The above solution was transferred into a Petri dish and dried at room temperature to get a thin film (relative humidity is about 54% at 25 °C). The specific capacitance, energy density, and power density were calculated using the following equations:^8,23,35^2
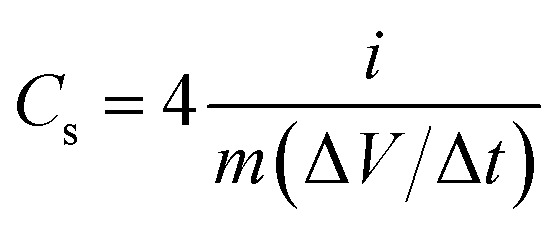
3
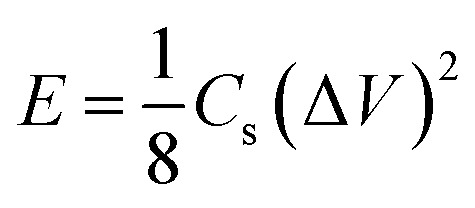
4
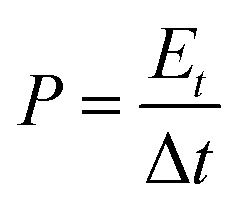
where *C*_s_ is the specific capacitance of the electrode materials (F g^−1^), *i* is the applied current (A), Δ*t* is the discharge time (s), *m* is the total mass of the active material on both electrodes, Δ*V* is the operating potential window (V), *E* is the energy density (Wh kg^−1^), and *P* is the power density (W kg^−1^). The power density and energy density are calculated by choosing the potential region from 0 to 1.5 V in the total potential window of −1.5 V to +1.5 V of the two-electrode symmetric system.

## 3. Results and discussion

**Scheme 1:**
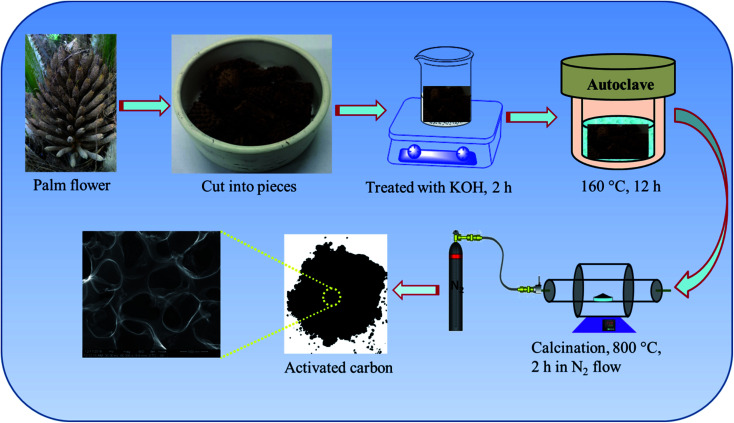
Hydrothermal method of preparing the nitrogen doped activated carbon samples from palm flowers.

The synthesis procedure of nitrogen doped activated carbon from palm flowers is illustrated in Scheme 1. It is known that the nitrogen doped carbons obtained from bio-waste materials and by other chemical synthesis methods are often quite disordered which can be functionalized for various applications.^29–35^ The hydrothermal method is regarded as one of the simplest and most versatile procedures providing a uniform reaction environment compared to conventional heating methods. The nitrogen content, surface chemistry and porous structure of carbon materials can also be tuned by hydrothermal treatment. In our study, we have adopted the hydrothermal method to prepare high surface area porous carbon from palm flower biomass. In the first step, we have used 2 M KOH as an activating agent to introduce micro/meso-porous structures into the carbon matrix obtained from palm flowers. In the second step, the surface area and porosity of carbons are tuned by varying the temperature of activation at 700, 800 and 900 °C in the flow of nitrogen. We have also synthesized activated carbon from palm flowers by a direct heat treatment method without hydrothermal treatment. In this case the BET surface area of activated carbon is 164 m^2^ g^−1^, which is five-fold less than that of the activated carbon prepared *via* a hydrothermal route followed by calcination. The hydrothermal route followed by pyrolysis in flowing nitrogen gas gave us the best carbon morphology and texture for charge storage application.

The powder X-ray diffraction pattern and nitrogen sorption isotherms of the activated carbons are shown in Fig. S1.† The BET surface areas of activated carbon samples are presented in Table S1.† The table shows that the specific surface area of the activated carbons increased from 610 to 796 m^2^ g^−1^, and the carbon synthesized at 800 °C exhibits the largest surface area. In order to find out the effect of KOH concentration, we have carried out the activation at three different molar concentrations of KOH (1, 2 and 3 M), while fixing the carbonization temperature at 800 °C. The corresponding powder X-ray diffraction patterns and nitrogen sorption isotherms are given in Fig. S2.† The activated carbon synthesized using 2 M KOH solution exhibits clearly a high specific surface area (796 m^2^ g^−1^). Since the double layer capacitor of any carbon sample depends on the surface area of the electrode material, we have optimized the concentration of KOH as 2 M and carbonization temperature at 800 °C to achieve the best double layer capacitor performance. During the activation process, the chemical reaction between KOH and carbon material typically consists of the following steps:^13,44^

5 4KOH + C → K_2_CO_3_ + K_2_O + 2H_2_

6 K_2_CO_3_ + 2C → 2K + 3CO

The K_2_CO_3_ generated in step 1 causes gasification of amorphous carbon to CO and yields K as shown in step 2, which subsequently intercalates in to the carbon lamella, leading to the formation of pores.

**Fig. 1:**
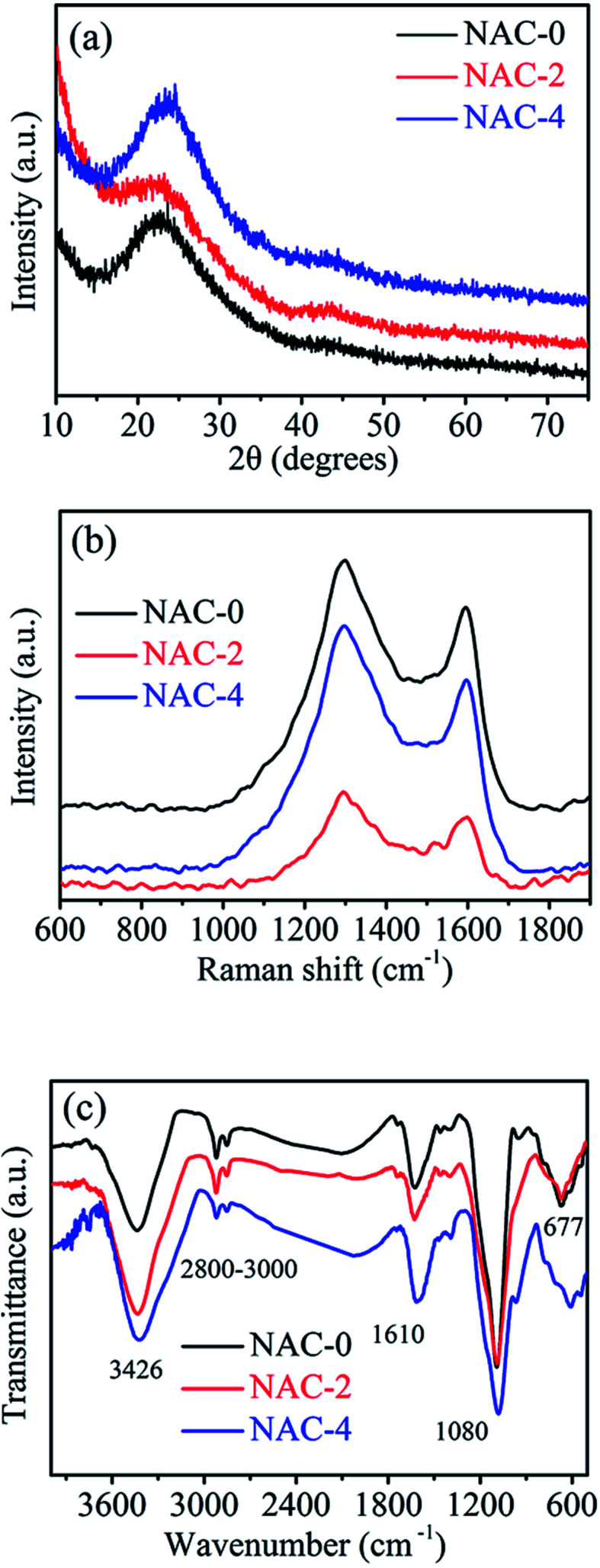
(a) XRD pattern, (b) Raman spectra and (c) FT-IR spectra of nitrogen doped activated carbons (NACs).

However, in order to improve the electrochemical performance of activated carbons towards charge storage, we have disturbed the carbon matrix by doping different wt% of nitrogen in it. The nitrogen content is tuned by varying the weight ratio of palm flower/melamine, where melamine is the precursor for nitrogen. The amounts of carbon, nitrogen and oxygen present in different nitrogen doped activated carbons are estimated by CHN analysis and given in Table 1. The wide angle powder X-ray diffraction pattern of the as synthesized NACs is shown in Fig. 1a. The broad peaks around ∼23° and 43° confirm the presence of disordered amorphous carbon. As compared to NAC-0 and NAC-4, the peak intensity of NAC-2 is weaker, indicating a lower degree of graphitization, which is further confirmed by the Raman spectra. Raman scattering measurements are carried out to estimate the degree of functionalization and structural evolution of NAC. Raman spectra of NAC-0, NAC-2 and NAC-4 are compared in Fig. 1b. All samples show two strong peaks at around 1298 and 1600 cm^−1^, which are indexed to D band and G band vibrations, respectively. The D band corresponds to the vibration of disordered carbon, while the G band reflects the bond stretching of ordered sp^2^ carbon. The relative intensity ratio of D band to G band (*I*_D_/*I*_G_) reveals the degree of graphitization as well as degree of defects on the carbon network. The *I*_D_/*I*_G_ ratio of NAC-2 is 1.35, which is significantly larger than that of NAC-4 (1.28) and NAC-0 (1.22), revealing that the presence of N-content would increase the degree of disorder in the activated carbon structure.^5,19^ The disorder in the N-doped carbon structures is due to the KOH activation in the presence of melamine. This result is consistent with the powder XRD pattern as well. In addition, the large value of *I*_D_/*I*_G_ ratio indicates the amorphous structure of NAC. However, the presence of a large number of defects in NAC-2 is highly advantageous for diffusion and adsorption of electrolyte ions inside the electrode materials and favourable to better charge storage performance. In order to throw light on the chemical nature of the nitrogen present in the carbon materials and their surface properties, FTIR spectra are recorded as displayed in Fig. 1c. The characteristic broad band at 3426 cm^−1^ is attributed to N–H/O–H stretching vibration. The two small peaks around 2800–3000 cm^−1^ are assigned to symmetric/asymmetric C–H stretching vibration and the peak at 1610 cm^−1^ is assigned to the C=C stretching vibration. The peaks at 1080 and 677 cm^−1^ are ascribed to C–N/C–O and out-of-plane C–H/N–H stretching vibration, respectively.^16,19^ It is noteworthy that the relative intensity of the peak at 3426 cm^−1^ in the NAC-0 sample is reduced significantly compared to those of NAC-2 and NAC-4 samples, which clearly indicates less amount of nitrogen present in the sample. Thermogravimetric analyses of activated carbons under an air atmosphere are shown in Fig. S3.† The small weight losses of activated carbons below 100 °C are due to the release of physisorbed water. The major weight losses seen in the temperature range of 390 to 630 °C from NAC-0, NAC-2 and NAC-4 samples are about 38%, 58% and 44%, respectively. These losses are ascribed to the decomposition of the carbon skeleton and oxygen/nitrogen-containing functional groups.^22^ The sample NAC-2 shows the highest weight loss.

**Fig. 2:**
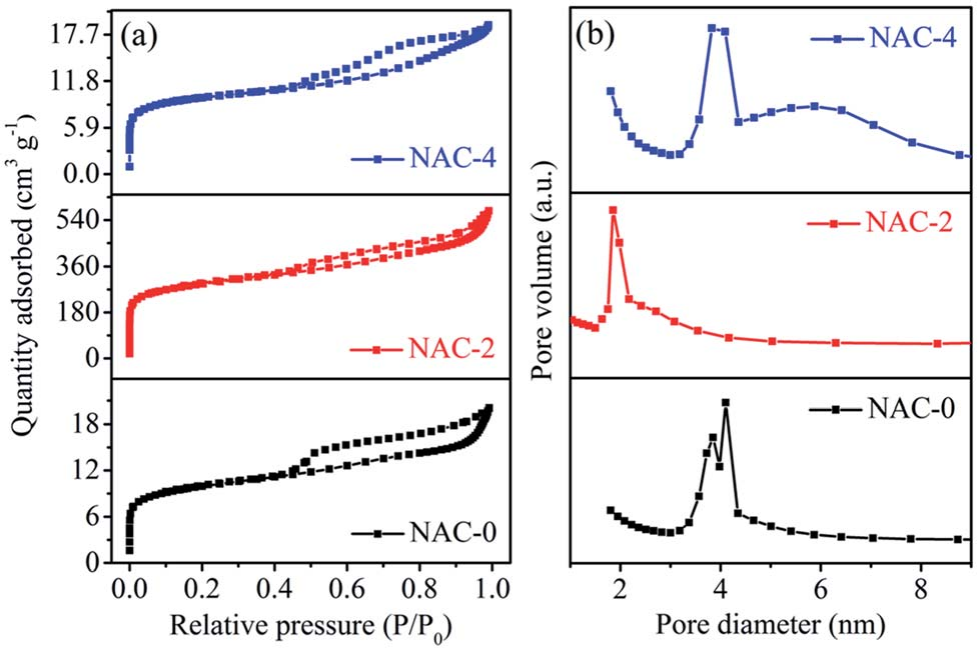
(a) BET adsorption–desorption profiles and (b) pore size distribution curves of N-doped activated carbons (NACs).

**Fig. 3:**
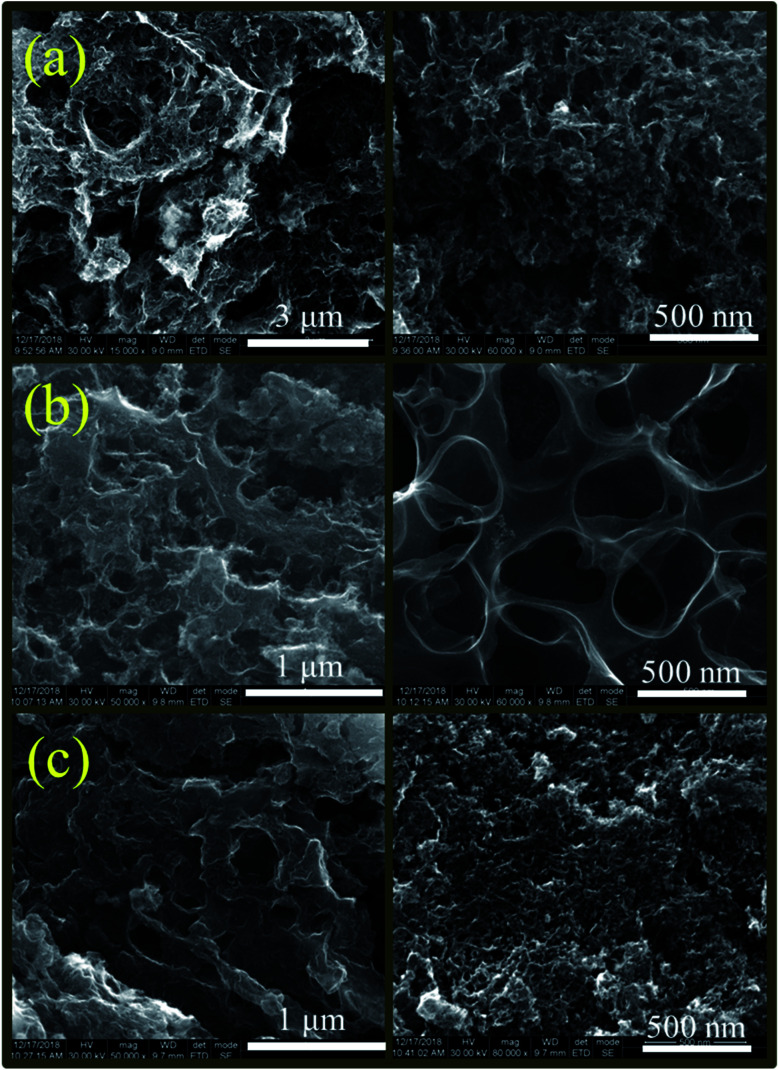
HR-SEM images of (a) NAC-0, (b) NAC-2 and (c) NAC-4 samples.

**Fig. 4:**
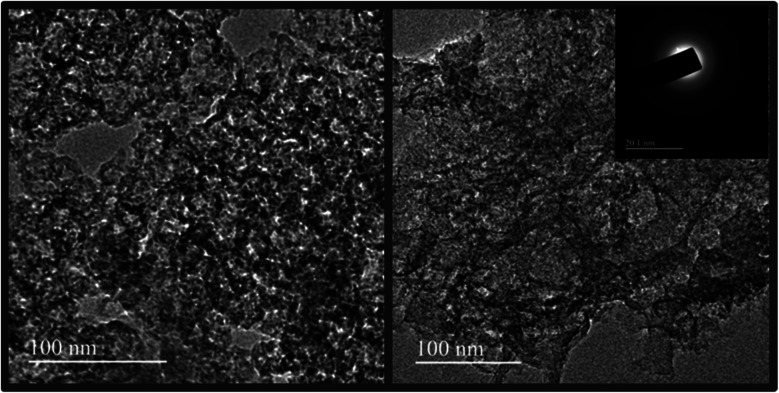
HR-TEM images of NAC-2. The inset shows the SAED pattern.

**Fig. 5:**
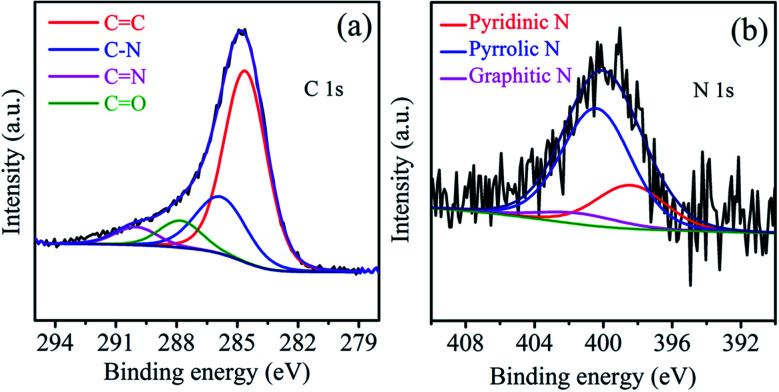
XP spectra of (a) C 1s and (b) N 1s regions of the NAC-2 sample.

The rich porosity of NACs is confirmed by N_2_ adsorption–desorption isotherms (Fig. 2). The N_2_ sorption isotherms of all samples exhibit a characteristic type IV isotherm with a distinct hysteresis loop H4, signifying mesoporous nature of NACs. The N_2_ uptake exhibiting type H4 hysteresis in the *P*/*P*_0_ range of 0.4–1.0 signifies the existence of narrow slit-shaped mesopores.^45^ The BET surface area and pore size of NAC-0, NAC-2 and NAC-4 are summarized in Table 1. The specific surface area of NAC-2 is 1054 m^2^ g^−1^, which is higher than that of NAC-0 and NAC-4 samples. The BET surface area of activated carbon increases with nitrogen doping and then decreases when an excess amount of nitrogen doping occurs in the carbon matrix. Excess doping of nitrogen in carbon can cause pore expansion.^19,20^ However, when the amount of nitrogen functional groups increases from 2 wt% to 4 wt% on the carbon surface, the structure of the carbon network seems to collapse, distorting the original structure. At 4 wt% nitrogen doping, some of the active sites of NAC carbon are blocked by excess amount of nitrogen functional groups, which originated from bulky melamine, interrupting the interconnections of the graphitic structure which leads to the collapse of the pore structure. The pore size distribution curves in Fig. 2b reveal that NAC-2 has well defined micro-pores while NAC-0 and NAC-4 contain mesopores. A structural change to activated carbon is observed while doping nitrogen atoms in it. The 2 wt% nitrogen doped activated carbon shows a smooth surface with distinct microporosity. However, the surface of the 4 wt% nitrogen doped sample is partially perforated or eroded, creating mesoporosity in the carbon network. The high surface area and well defined micro/meso-pore structures facilitate the electrolyte diffusion and ion transport into the electrode materials, thus enhancing the charge storage characteristics of NAC-2.^46^ The microstructures of the as-prepared nitrogen doped activated carbons are further studied by HR-SEM and TEM analysis. The HR-SEM images in Fig. 3 show the porous nature of the NAC samples subjected to the KOH chemical activation. The higher magnification SEM image of NAC-2 in Fig. 3b is distinctly different from that of the other two samples. It clearly shows the interconnected porous network consisting of numerous micro/mesopores which are crucial for faster adsorption and diffusion of ions from the electrolyte solution. The TEM images of NAC-2 in Fig. 4 show rough micro- and nanopore structures of the carbon framework (Fig. 4). The selected-area electron diffraction (SAED) pattern suggests the amorphous nature of the carbon structure (inset in Fig. 4). The surface chemical compositions of the NAC-2 sample are analyzed by XPS as shown in Fig. 5. Fig. 5a is the deconvoluted spectrum of C 1s which shows C–C (284.6 eV), C–N (285.9 eV), C=O (287.8 eV) and C=N (290.0 eV) groups present in the NAC-2 sample. The N 1s spectrum in Fig. 5b consists of three peaks corresponding to pyridinic nitrogen (398.6 eV), pyrrolic nitrogen (400.4 eV) and graphitic nitrogen (401.5 eV). The conductivities of activated carbons are measured by two probe techniques. As expected, introduction of the nitrogen group into the carbon matrix improves the conductivity of the activated carbons. The calculated conductivity of NAC-0 is 0.33 S m^−1^. The nitrogen doped samples NAC-2 and NAC-4 show higher conductivity values of 2.01 S m^−1^ and 1.87 S m^−1^, respectively. It is observed that NAC-4 has higher nitrogen content than NAC-2 but exhibits lesser conductivity. The NAC-2 sample seems to have a higher content of graphitic nitrogen which is the reason for showing slightly higher conductivity. The nitrogen atoms in the cyclic carbon framework impart a rich electronic structure, different from pure carbon, to the NAC-2 sample which is expected to enhance the pseudocapacitance by redox reactions as well as an increase in the conductivity of carbon materials.^47^

**Fig. 6:**
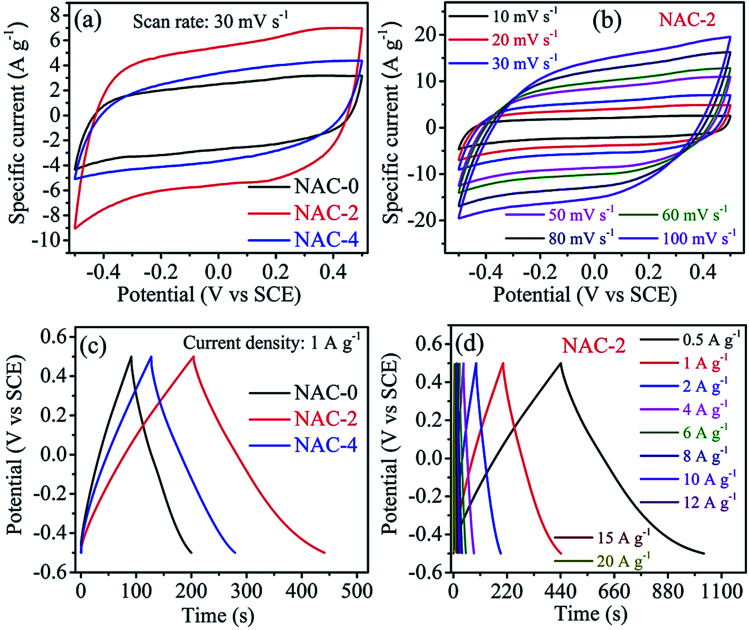
CV profiles of (a) NAC-0, NAC-2 and NAC-4 at 30 mV s^−1^ and (b) the NAC-2 electrode at different scan rates, (c) galvanostatic charge–discharge profile of NAC-0, NAC-2 and NAC-4 at 1 A g^−1^ current density, and (d) galvanostatic charge–discharge profiles of the NAC-2 electrode at different current densities.

Considering all the features of carbon samples discussed above, NACs are potential materials for fabricating miniscule devices. As a proof of concept, these materials are tested for supercapacitor applications in aqueous, non-aqueous and solid gel electrolytes employing both two and three electrode setups. The supercapacitive performances are evaluated by CV, GCD and EIS techniques. Initially the three electrode configuration has been chosen to carry out electrochemical studies using 0.1 M aqueous H_2_SO_4_. Fig. 6a shows the comparative CV tests of NAC-0, NAC-2 and NAC-4 at a scan rate of 30 mV s^−1^. All the curves exhibit typical rectangular curves from −0.5 to 0.5 V, which implies that the charge stored is essentially by the electrochemical double layer mechanism with a small contribution coming from the pseudocapacitance due to oxygen/nitrogen groups present on NACs.^48,49^ The integral area under the curve for NAC-2 is much higher than that of NAC-0 and NAC-4 samples, indicating higher capacitance performance of NAC-2 by improved ion diffusion/adsorption processes. The NAC-2 sample is expected to offer the highest capacitance due to large surface area which is hereby confirmed by the CV test. The rate performance of NAC-2 is shown in Fig. 6b, which displays good shape preservation of CV curves even at high scan rates, suggesting good charge propagation within the electrode. Again the area of the CV curve increases with increasing scan rate, which is related to diffusion of the active ions in micro/meso-porous structures. At high scan rates, the ionic mobility of electrolyte ions becomes faster, thereby increasing the current response. The CV profiles of NAC-0 and NAC-4 at different scan rates are shown in Fig. S4.†

**Fig. 7:**
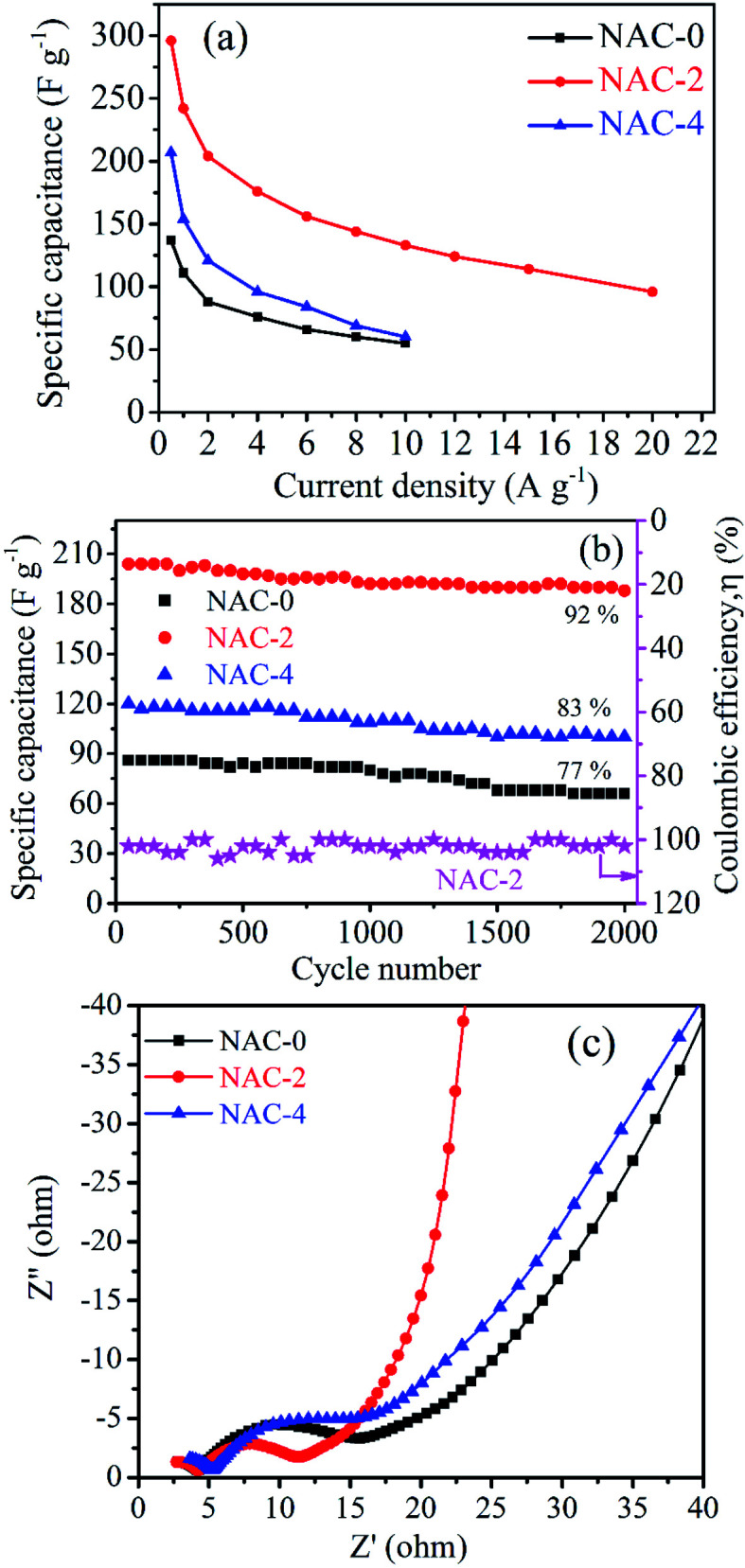
(a) Specific capacitance as a function of current densities, (b) specific capacitance as a function of cycle number and (c) Nyquist plots of NAC-0, NAC-2 and NAC-4 electrodes.

The galvanostatic charge/discharge curves of NAC-0, NAC-2 and NAC-4 samples at a current density of 1 A g^−1^ are shown in Fig. 6c. It is observed that the GCD curves of all NAC samples exhibit a triangular shape with a quasi-symmetrical charge/discharge curve, indicating the electrochemical double layer performance. The comparative GCD curves of all carbon samples (Fig. 6c) show that NAC-2 is taking longer time for accomplishing the charge/discharge curve than NAC-0 and NAC-4 samples. This represents an enhanced double layer capacitance performance of the NAC-2 sample. The voltage drops (at the start of a discharge curve) of NAC-0, NAC-2 and NAC-4 samples at a current density of 0.5 A g^−1^ are 0.021, 0.012 and 0.019 V, respectively. The lowest voltage drop value of NAC-2 indicates that it has the highest electrical conductivity and low equivalent series resistance (ESR) in the assembled capacitor.^19,48^ The GCD profiles of NAC-2 at different current densities are shown in Fig. 6d. Here the shape of the charge/discharge curve is quasi-symmetrical, indicating that the charge storage mechanisms contribute to the effect of hetero atom (N and O elements) doping. The GCD curves of NAC-0 and NAC-4 samples are displayed in Fig. S4.† The specific capacitance values of electrode materials are calculated from GCD curves using eqn (1). It is observed that the specific capacitance of NAC-2 decreased gradually from 296 to 96 F g^−1^ with a gradual increase in current density from 0.5 to 20 A g^−1^. This decrease in specific capacitance is due to the fact that large current restricts the diffusion and migration rates of electrolyte ions within the electrode material, and therefore, only a part of the material remains active. The specific capacitance of the NAC-2 electrode at 0.5 A g^−1^ is about 296 F g^−1^, which is much higher than that of the activated carbon prepared from coconut shells (100 F g^−1^) even though it has high specific area (1340 m^2^ g^−1^). This implies that along with surface area there are other factors such as surface modification, pore diameter and conductivity which play a significant role in enhancing the charge storage performance of carbons.^7,19,50^Fig. 7a shows the variation of specific capacitance at various current densities for all NAC electrode materials. The specific capacitance of NAC samples has been improved by about 2.3 times after nitrogen doping. However, the improvement is quite significant when N-doping is 2 wt% where the material seems to have optimal amounts of nitrogen functional groups in the carbon framework as well as sufficient conductivity and active sites favourable for ion adsorption. However, doping an excess number of nitrogen heteroatoms may decrease the conductivity with more resistive behavior and number of active sites in the carbon framework as well.^19^ We have tabulated the charge storage performance of carbons derived from various biomass sources in Table 2. The table shows that the specific capacitance of NAC-2 is higher than or comparable to that of other bio-waste derived carbons. The long term electrochemical stability of NAC-2 is examined by continuous 2000 GCD cycles at a current density of 2 A g^−1^. The capacitance retention and the coulombic efficiency of the NAC-2 sample, respectively, are about 92% and 98% after 2000 GCD cycles. However, NAC-0 and NAC-4 samples retain 77% and 83% of their initial capacitance after 2000 GCD cycles, respectively, which indicate excellent pore accessibility and long term electrochemical stability of the nitrogen-doped carbon materials (Fig. 7b).

**Table 2:** Comparison of charge storage performance of different biomass carbon materials

Carbon derived from	Type of carbon product	*S*_BET_ (m^2^ g^−1^)	Specific capacitance (F g^−1^)	Energy density (Wh kg^−1^)	Power density (W kg^−1^)	Ref.
Sugarcane	Porous carbon aerogel	1892	268.4 at 2 mV s^−1^	19.74	500	5
Coal	N,O-co-doped porous carbon/carbon nanotube composite	2164	287 at 0.2 A g^−1^	5.3	500	14
Gelatin biomolecule	N-doped mesoporous carbon	804	254 at 1 mA cm^−2^	—	—	15
Rice straw	Activated porous carbon	1007	332 at 0.5 A g^−1^	17.4	126	22
Pecan nutshell waste	Activated carbon	1085	150 at 5 mV s^−1^	10	1000	25
Human hair	N,S-doped activated hydrothermal carbons	849	264 at 0.25 A g^−1^	—	—	26
Silk	N-doped carbon nanosheet	2494	242 at 0.1 A g^−1^	90	875	27
Banana fibers	Activated carbon	1097	74 at 0.5 A g^−1^	—	—	30
Jute stick	Nanoporous carbon	2393	282 at 0.5 A g^−1^	4.2	12 420	32
Wheat straw	N-doped hierarchical porous carbon	892	275 at 0.2 A g^−1^	—	—	34
Bean curd	N-doped hierarchical porous carbon	2180	284 at 0.1 A g^−1^	12	50	35
Sunflower stalk	Activated carbon	1505	365 at 1 A g^−1^	35.7	989	44
Coconut shell	Activated carbon	1340	100	—	—	50
Palm flower	N-doped activated carbon	1054	296 at 0.5 A g^−1^	5.5	821.2	This work

Electrochemical impedance spectroscopic (EIS) study has been carried out on NAC-0, NAC-2 and NAC-4 samples at an applied bias potential of −0.4 V and the corresponding Nyquist plots are presented in Fig. 7c. There are two distinct parts in the plots, a vertical line in the low frequency region and a semicircle in the high frequency region. The solution resistance (*R*_s_) can be estimated from the intercept on the real axis in the high frequency region. Accordingly the *R*_s_ values obtained from the plots are ∼2.5, 2.5 and 2.9 Ω for NAC-0, NAC-2 and NAC-4 electrodes, respectively. The diameter of the semicircle in the high frequency region refers to the charge transfer resistance (*R*_ct_), which implies the migration of ions at the interface between the electrolyte and electrode surface. However, the smaller diameter of the semicircle in the spectra indicates lower *R*_ct_. Fig. 7c shows that the NAC-2 sample has a semicircle with the smallest diameter. The corresponding *R*_ct_ values of NAC-0, NAC-2 and NAC-4 samples are also measured to be 9.6, 6.2 and 8.1 Ω, respectively. The NAC-2 has the smallest *R*_ct_ value which implies that it has a better electrically conductive network than the other NAC samples. The vertical straight line in the low frequency region corresponds to the Warburg impedance, which is a measure of the diffusion of ions through the active electrode material. The vertical straight line of the NAC-2 electrode is steeper than the others, which indicates good capacitive behavior of the NAC-2 sample. It is therefore confirmed using the three-electrode system that an appropriate amount of hetero atom doping in the carbon matrix can in principle enhance the active sites as well as conductivity of carbon materials and boost their charge storage performance.

**Fig. 8:**
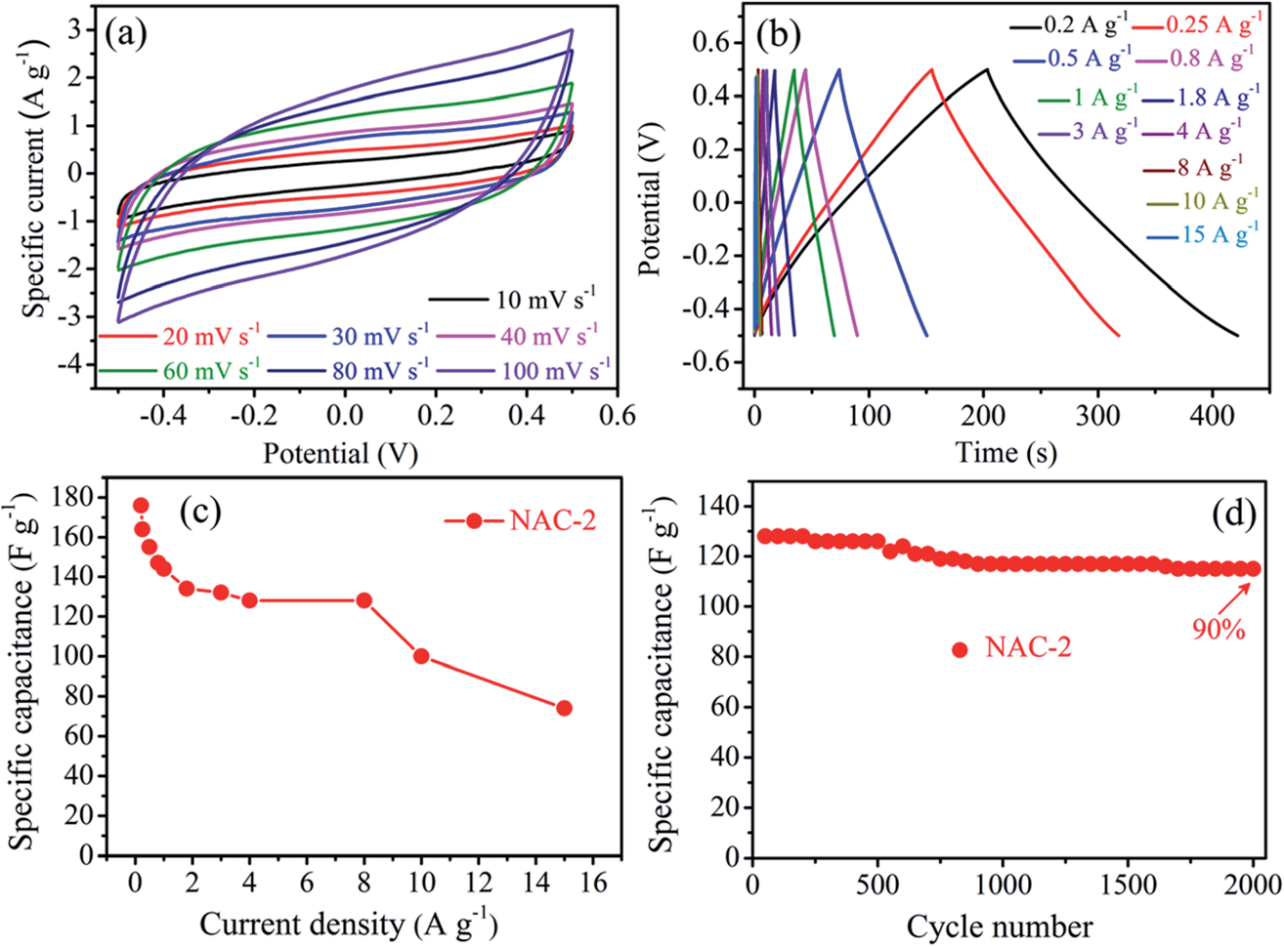
(a) CV profiles of the NAC-2 material electrode at different scan rates, (b) GCD profiles of the NAC-2 electrode at different current densities, (c) specific capacitance of NAC-2 as a function of current densities, and (d) specific capacitance as a function of cycle number for the NAC-2 electrode.

In order to evaluate the practical applications of the NAC-2 material for supercapacitors, equal amounts of active material are coated on both the electrodes which were assembled in a symmetrical two-electrode system with carbon cloth as the current collector. The device fabricated is tested in 0.1 M H_2_SO_4_ aqueous electrolyte in the potential range of −0.5 to 0.5 V. The CV profile of NAC-2 at various scan rates is shown in Fig. 8a. All CV curves are in a rectangular shape, confirming perfect capacitive behavior of the NAC-2 electrode. Moreover, with increasing the scan rates from 10 to 100 mV s^−1^, the current density increased continuously, indicating good rate capability of the NAC-2 material. The GCD curves of NAC-2 at various current densities are shown in Fig. 8b. NAC-2 exhibits symmetric triangular GCD curves due to high reversibility between charge/discharge processes. The specific capacitance of NAC-2 employed in the symmetric capacitor is calculated by eqn (2) and its value is 176 F g^−1^ at a current density of 0.2 A g^−1^. Fig. 8c shows the specific capacitance behaviour of NAC-2 at various current densities. The cycling test of the fabricated symmetric device in Fig. 8d shows that NAC-2 retains 90% of its initial capacitance after 2000 cycles at a current density of 8 A g^−1^, indicating its worthiness for electrochemical devices. In addition, the GCD curves of 1^st^ and 2000^th^ cycles are shown in Fig. S5,† which also prove that the electrochemical performance of NAC-2 is excellent. Fig. S6† shows the Nyquist plot of the NAC-2 electrode in the frequency range from 0.01 Hz to 100 kHz. An equivalent circuit model appropriate to fit the EIS data of the NAC-2 electrode is obtained using ZsimpWin software (inset of Fig. S6†). By fitting this model into impedance data, the simulated values of *R*_s_, *R*_ct_, and *Z*_w_ are determined to be 3.50, 0.31 and 0.16 Ω, respectively. The low values of *R*_s_, *R*_ct_, and *Z*_w_ confirm that the fabricated symmetric cell exhibits low electrolyte resistance and low contact resistance between the active material and the current collector, and hence increase the capacitance of the NAC-2 electrode.

The energy density of a capacitor is directly proportional to the specific capacitance and square of its operating voltage. Therefore, either specific capacitance or operating voltage of a capacitor has to be improved for its commercialization. Commonly used aqueous electrolytes have limitations in their working potentials which cannot go beyond the water decomposition potential of 1.2 V which is a bottleneck for practical applications. Several attempts have been made with limited success to enhance the specific capacitance of materials by designing porous carbons, metal oxides/sulfides, and composite materials and also increasing the cell voltage by designing asymmetric devices.^15,19,46,51–53^ Recently, organic electrolytes which are stable up to 2 to 3 V, and ionic liquids up to 5 V have been employed to improve the energy density of supercapacitors.^54^ In this context, we have tested NAC-2 material in a 2-electrode symmetric cell by employing [EMIM][ESO_4_] ionic liquid electrolyte. The charge storage property of carbon materials is strongly related to the nature of the electrolyte. Usually, the accessibility of electrolyte ions at the electrode/electrolyte interface is different for aqueous and ionic liquid based electrolytes. Ionic liquids are non-corrosive unlike acidic electrolytes. They act as good surfactants preventing the agglomeration of carbon particles during continuous charge–discharge cycles. Hence, presence of the ionic liquid weakens the face-to-face van der Waals forces between carbon layers and facilitates uniform distribution of carbon particles, providing good conductivity and accessibility of the carbon network to the electrolyte.

**Fig. 9:**
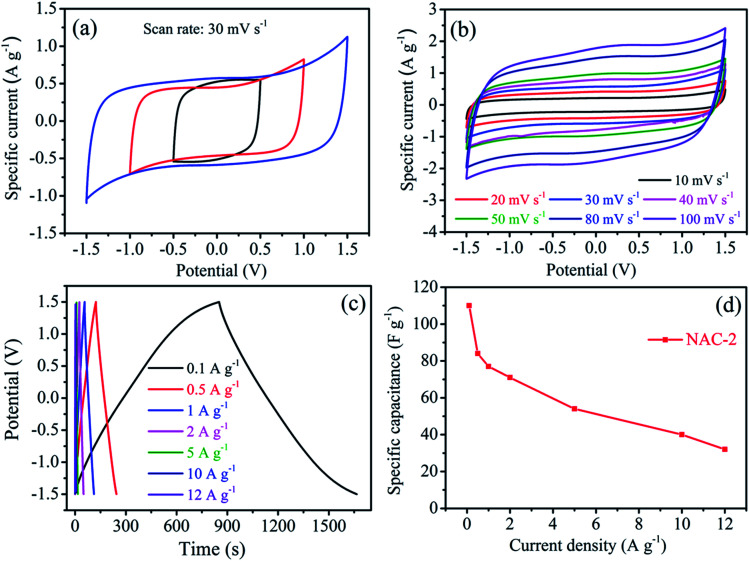
CV curves of the NAC-2 electrode (a) at different potential windows, (b) at different scan rates, (c) GCD curves of NAC-2 at different current densities in a two electrode symmetric device configuration using [EMIM][ESO_4_] ionic liquid electrolyte, and (d) specific capacitance of the NAC-2 electrode as a function of current density.

**Fig. 10:**
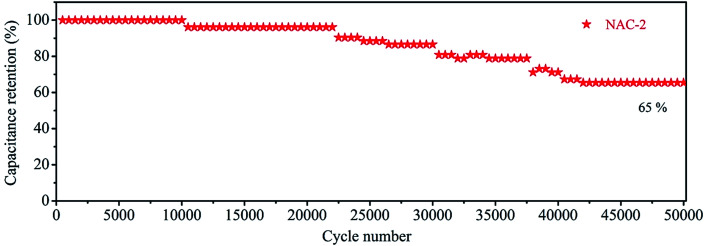
Cycling performance of the symmetric supercapacitor device in [EMIM][ESO_4_] electrolyte.

**Fig. 11:**
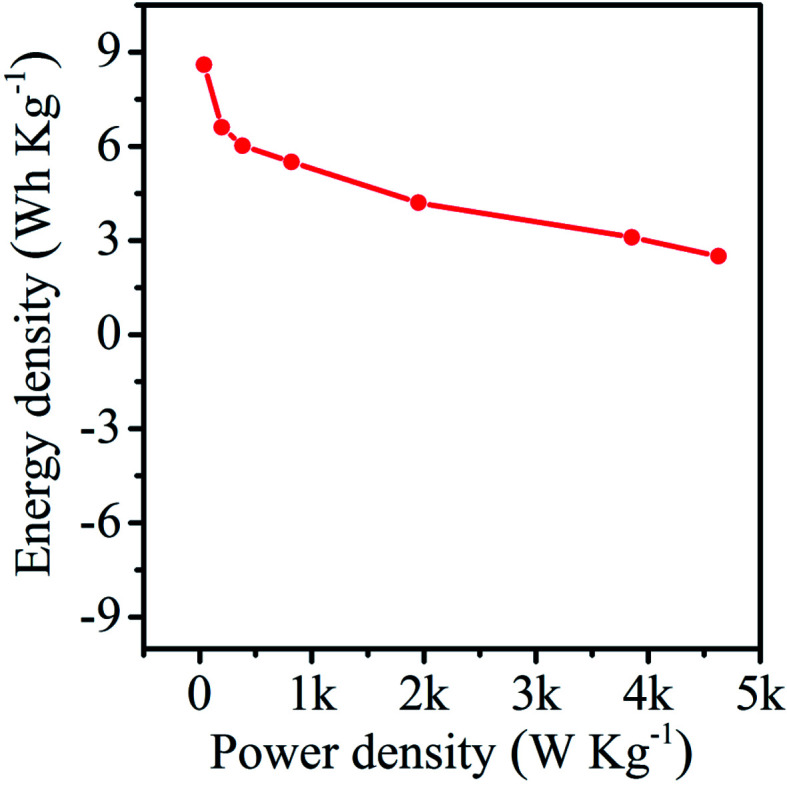
Ragone plot for the symmetric supercapacitor device fabricated using NAC-2 material and [EMIM][ESO_4_] ionic liquid electrolyte.

The CV curves of the NAC-2 symmetric cell at 30 mV s^−1^ scan and different potential windows are shown in Fig. 9a. This study confirms the stability of the electrode up to 3 V. Fig. 9b displays the CV curves of NAC-2 at different scan rates ranging from 10 to 100 mV s^−1^. The CV curves have a near rectangular shape even at a high scan rate of 100 mV s^−1^. This again confirms the good rate capability of the NAC-2 electrode. Typical GCD curves, and the graph of specific capacitance *versus* current density are shown in Fig. 9c and d. The specific capacitance of the NAC-2 electrode at 0.1 A g^−1^ is about 110 F g^−1^, which is comparable to the recently reported value.^22^ All GCD curves exhibit linear behavior with a symmetric triangle, indicating high coulombic efficiency of NAC-2. The stability of the NAC-2 electrode is evaluated by continuously repeating 50 000 GCD cycles at 5 A g^−1^ as shown in Fig. 10. The specific capacitance is almost constant up to 22 000 cycles, then drops by ∼30%, and remains constant from 40 000 to 50 000 cycles. The GCD curves of NAC-2 are recorded at different intervals of stability states, which are shown in Fig. S7.† These curves show excellent cycling stability in [EMIM][ESO_4_] ionic liquid electrolyte. The above results indicate that [EMIM][ESO_4_] ionic liquid and NAC-2 combination is favorable for energy storage devices. The Ragone plot that relates power density to the energy density is calculated from the GCD curves of the NAC-2 electrode in [EMIM][ESO_4_] ionic liquid electrolyte by eqn (3) and (4) and plotted in Fig. 11. The symmetric cell has the capability to deliver an energy density and power density of 8.6 Wh kg^−1^ and 38.9 W kg^−1^, respectively, at a current density of 0.1 A g^−1^ in the potential window of 1.5 V (two-electrode system). Similarly when the current density is increased from 0.1 to 12 A g^−1^, the energy density and power density are found to be 2.5 Wh kg^−1^ and 4627 W kg^−1^, respectively.

**Fig. 12:**
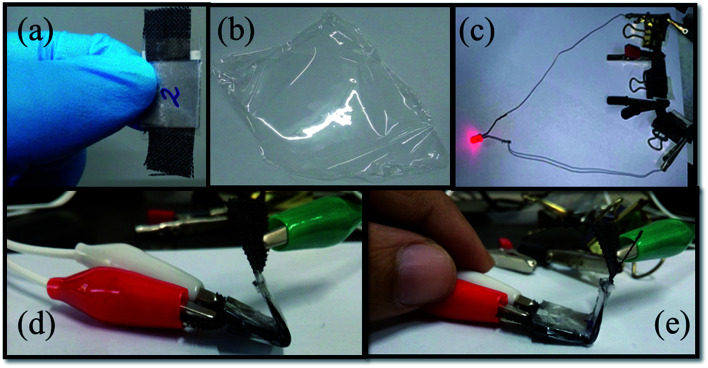
Photographic images of (a) the fabricated symmetric solid state capacitor device, (b) PVA/H_2_SO_4_ polymer electrolyte, (c) glowing LED light connected to three devices in series, and (d and e) demonstration of flexibility and bendability of the device.

**Fig. 13:**
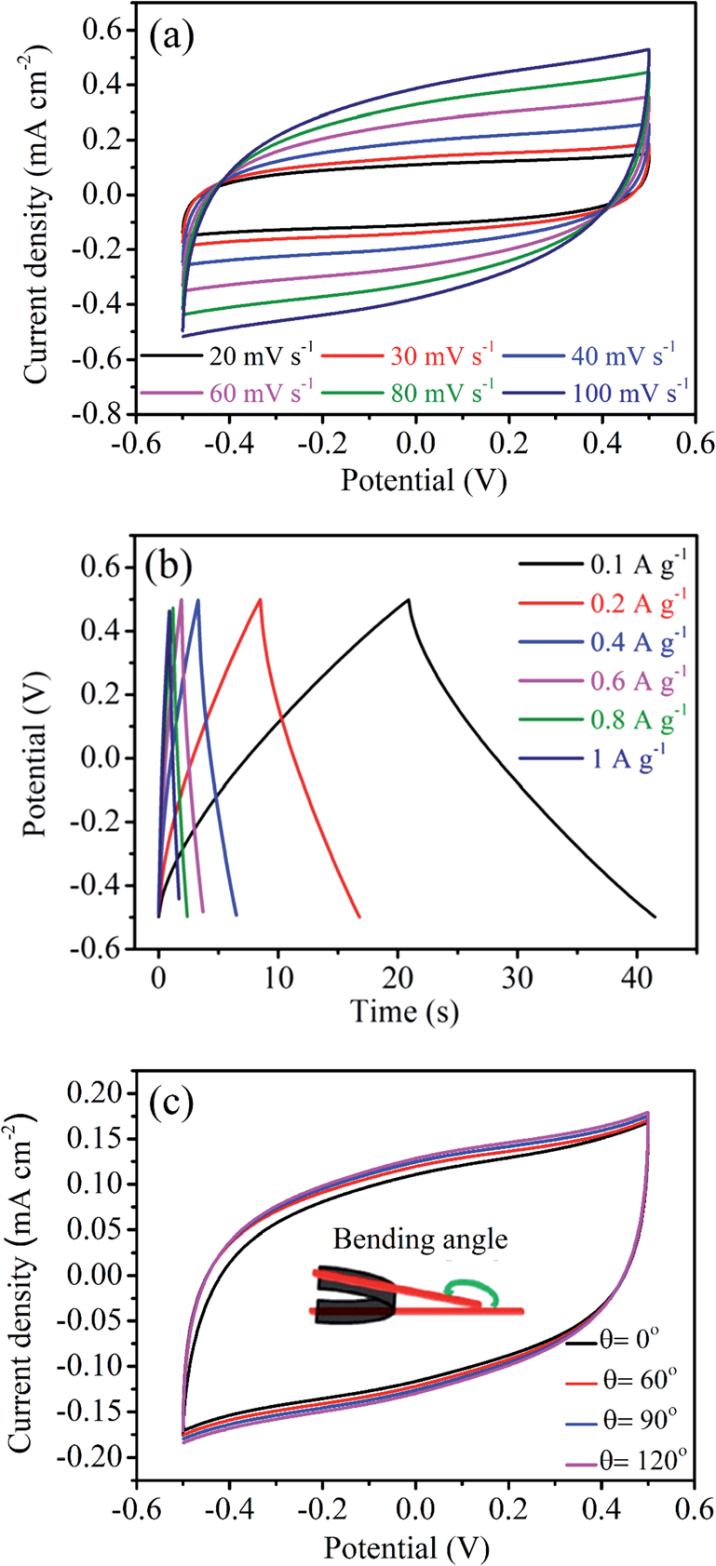
Electrochemical performance of the solid state symmetric device of the NAC-2 electrode using PVA/H_2_SO_4_ solid polymer electrolyte. (a) CV curves at different scan rates, (b) GCD curves at different current densities and (c) CV curves at different bending angles.

In the current scenario, microelectronic industries are driven by cheap, wearable, flexible, foldable and light weight electronic devices. A number of investigations have been reported on flexible supercapacitors,^55–57^ where current collectors and electrolytes are the key components.^58^ We have built a solid state flexible capacitor by choosing the NAC-2 material as an electrode, carbon cloth as the current collector, and 0.1 M H_2_SO_4_/PVA as the solid state electrolyte. The synthesis procedure of 0.1 M H_2_SO_4_/PVA polymer electrolyte is given in the Experimental section. A rationally designed NAC-2 fabricated flexible solid state symmetric capacitor is shown in Fig. 12. The optical image of three symmetric devices in series connection to glow a red light-emitting diode (LED) is shown in Fig. 12c. Fig. 13a shows the CV curves of the assembled flexible device at various scan rates from 20 to 100 mV s^−1^. The rectangular shape of the CV curves is maintained without any distortion even at high scan rate which suggests an excellent capacitive behavior of the device. The GCD curves of the flexible device at various current densities are shown in Fig. 13b. The shapes of these curves are triangular and symmetric, indicating good reversibility of charge/discharge processes. The photographic images of the fabricated flexible and bendable solid state device are shown in Fig. 12a–e. As expected, the estimated charge transfer resistance (*R*_ct_) from EIS measurements (Fig. S8†) is 3.68 Ω in 0.1 M H_2_SO_4_/PVA solid electrolyte which is higher than the *R*_ct_ value of 0.31 Ω in 0.1 M H_2_SO_4_ electrolyte. The CV profiles of the fabricated flexible solid state symmetric device are recorded at different bending angles to measure the effect of bending the device on capacitance as shown in Fig. 13c. The rectangular shape of the CV curves remains the same at different bending angles ranging from 0° to 120°. These measurements unequivocally confirm the mechanical flexibility of the electrode without affecting the charge storage capacity of the device. The similarity of the CV curves strongly supports the fact that bending the NAC-2 electrode does not disturb the electrical contact between the current collector and electrode material and the specific capacitance remains constant. We have demonstrated that environmental friendly N-doped activated carbons obtained from palm flower residue can serve as potential electrode materials for sustainable charge storage applications in supercapacitors.

## 4. Conclusions

In summary, activated carbons are extracted from palm flowers at different temperatures and KOH concentrations. Chemical activation of palm flowers with 2 M KOH at 800 °C gives rise to carbons with surface areas of ∼800 m^2^ g^−1^. Nitrogen doping into carbon produces activated carbons with surface areas of ∼1054 m^2^ g^−1^. The palm flower derived N-doped activated carbon electrodes are studied for supercapacitor applications in an aqueous, nonaqueous (ionic liquid) and flexible solid state membrane (0.1 M H_2_SO_4_/PVA) by fabricating a symmetric device. The micro- and meso-porous nature of NAC was confirmed by HR-SEM, HR-TEM and BET analysis. Among all the activated carbons, NAC-2 shows the highest specific capacitance of 296 F g^−1^ at 0.5 A g^−1^. In the laboratory experiment, a red LED is illuminated using three serially connected 3 V supercapacitor stacks. Using [EMIM][ESO_4_] ionic liquid as the electrolyte, the potential window of the symmetric device is extended up to 1.5 V (two-electrode system) and the device delivered a high energy of 8.6 Wh kg^−1^ at 0.1 A g^−1^ and 4627 W kg^−1^ at 12 A g^−1^, respectively. The most important result is that the device exhibited long term cycling stability over 50k cycles. The device can be bent without affecting its charge storage performance. The porous N-doped activated carbons obtained from palm flower residues are promising materials to fabricate environmental friendly, sustainable and flexible supercapacitors at low costs with good performance.

## Conflicts of interest

There are no conflicts to declare.

## Supplementary Material

NA-003-D1NA00261A-s001
