# Supplementary material for: A high energy flexible symmetric supercapacitor fabricated using N-doped activated carbon derived from palm flowers
Source: Nanoscale Adv. 2021 Aug 10;3(18):5417–29. doi: 10.1039/d1na00261a (PMC9417211; doi:10.1039/d1na00261a)
Supplement: NA-003-D1NA00261A-s001 [file NA-003-D1NA00261A-s001.pdf]

Electronic Supplementary Material (ESI) for Nanoscale Advances.
This journal is © The Royal Society of Chemistry 2021

High energy flexible symmetric supercapacitor fabricated using N-doped activated carbon derived from palm flower

Malaya K. Sahoo and G. Ranga Rao*

Department of Chemistry and DST-Solar Energy Harnessing Centre (DSEHC), Indian Institute of Technology Madras, Chennai 600036, India

Table S1 Surface areas of carbons extracted from palm flower residue using KOH solution and calcination at different temperatures.

Activation conditions of carbon [KOH] / calcination temperature	BET surface area ($m^2\ g^{-1}$)
2 M / 700 °C	786
2 M / 800 °C	796
2 M / 900 °C	610
1 M / 800 °C	625
2 M / 800 °C	796
3 M / 800 °C	492

*Corresponding author; Tel.: +91 44 2257 4226; Fax: +91 44 2257 4202; E-mail: grrao@iitm.ac.in (G. Ranga Rao).

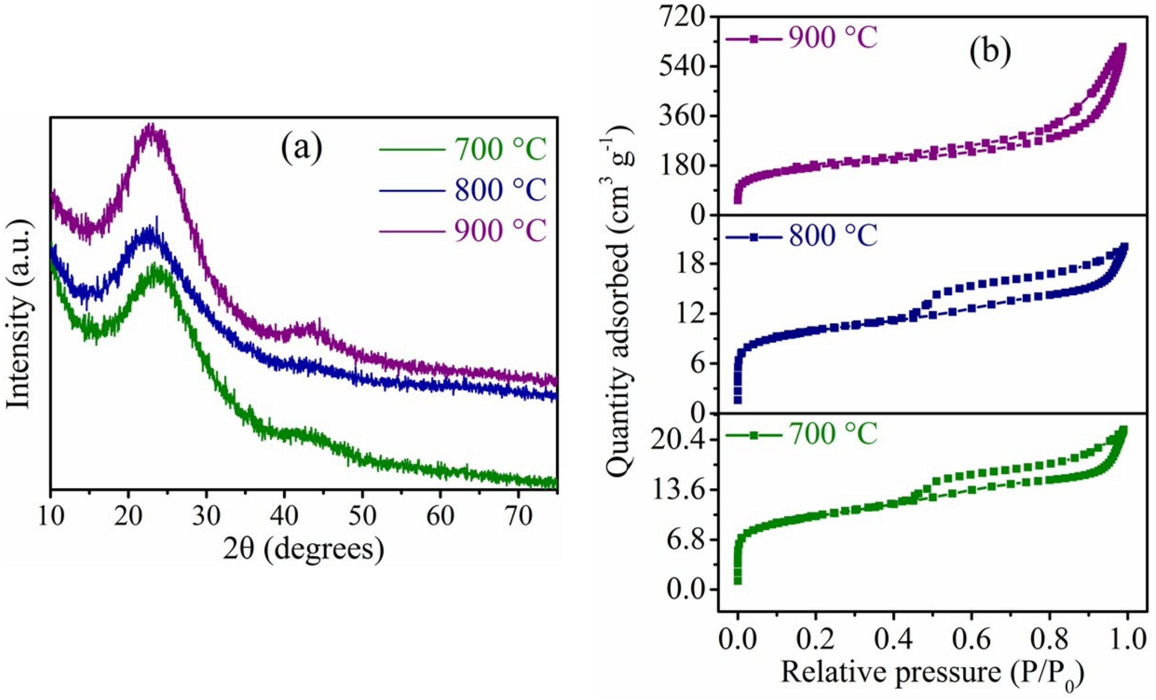

Fig. S1 (a) PXRD patterns and (b) BET isotherms of activated carbons obtained by activation of palm flower using 2 M KOH under autoclave conditions at 160 °C and calcined at different furnace temperatures in nitrogen flow.

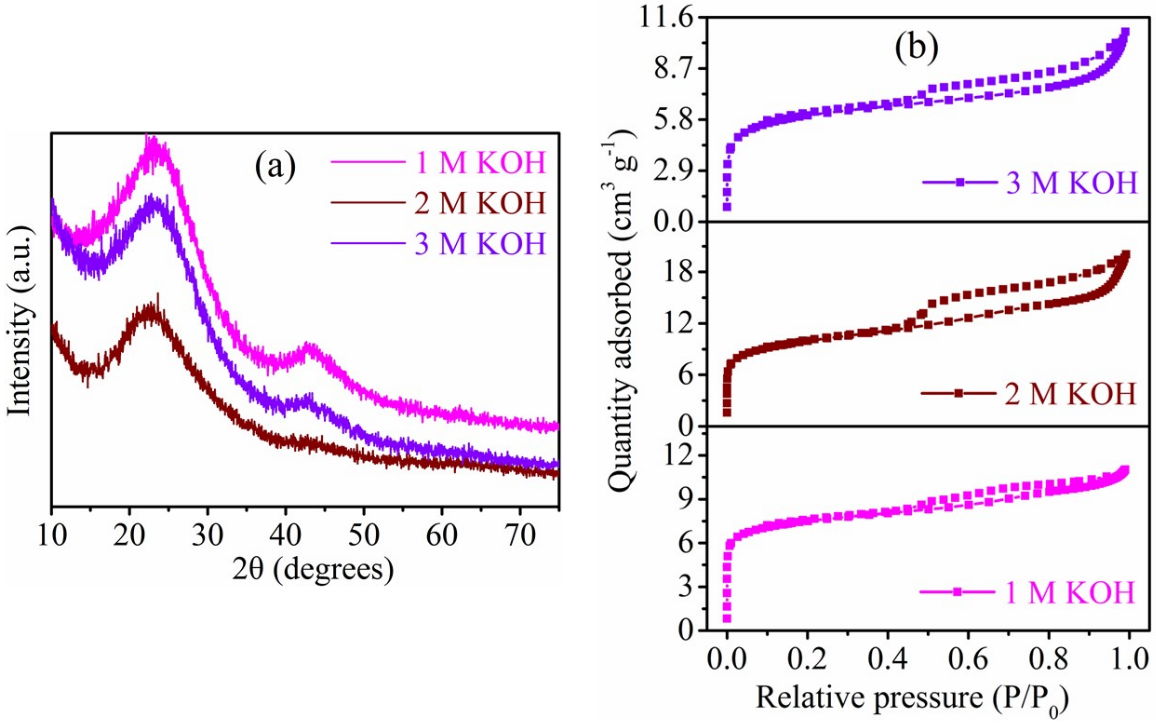

Fig. S2 (a) PXRD patterns and (b) BET isotherms of activated carbons obtained by activation of palm flower using 1, 2, and 3 M KOH under autoclave conditions at 160 °C and calcined at 800 °C fixed furnace temperature in nitrogen flow.

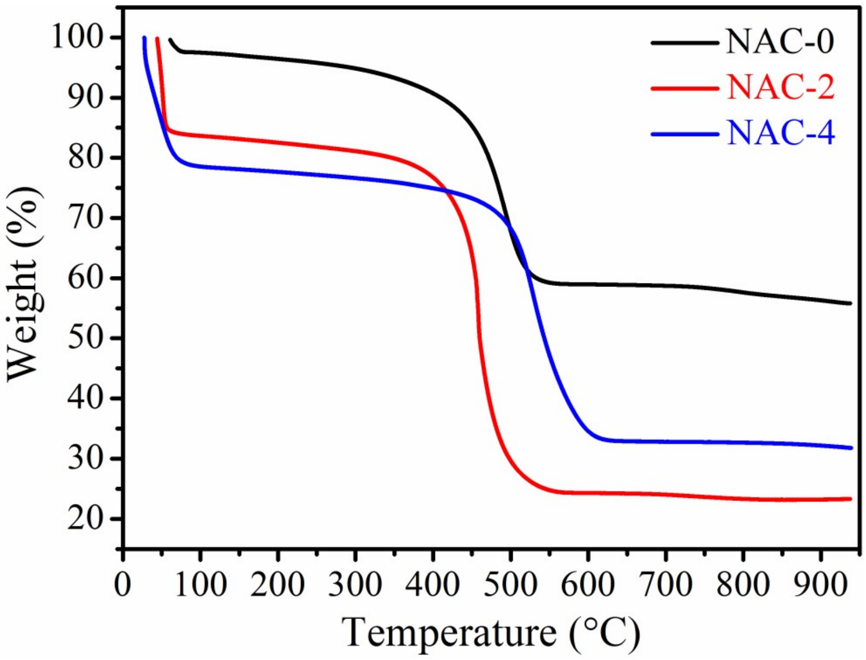

Fig. S3 TGA curves of nitrogen doped activated carbons recorded in air atmosphere.

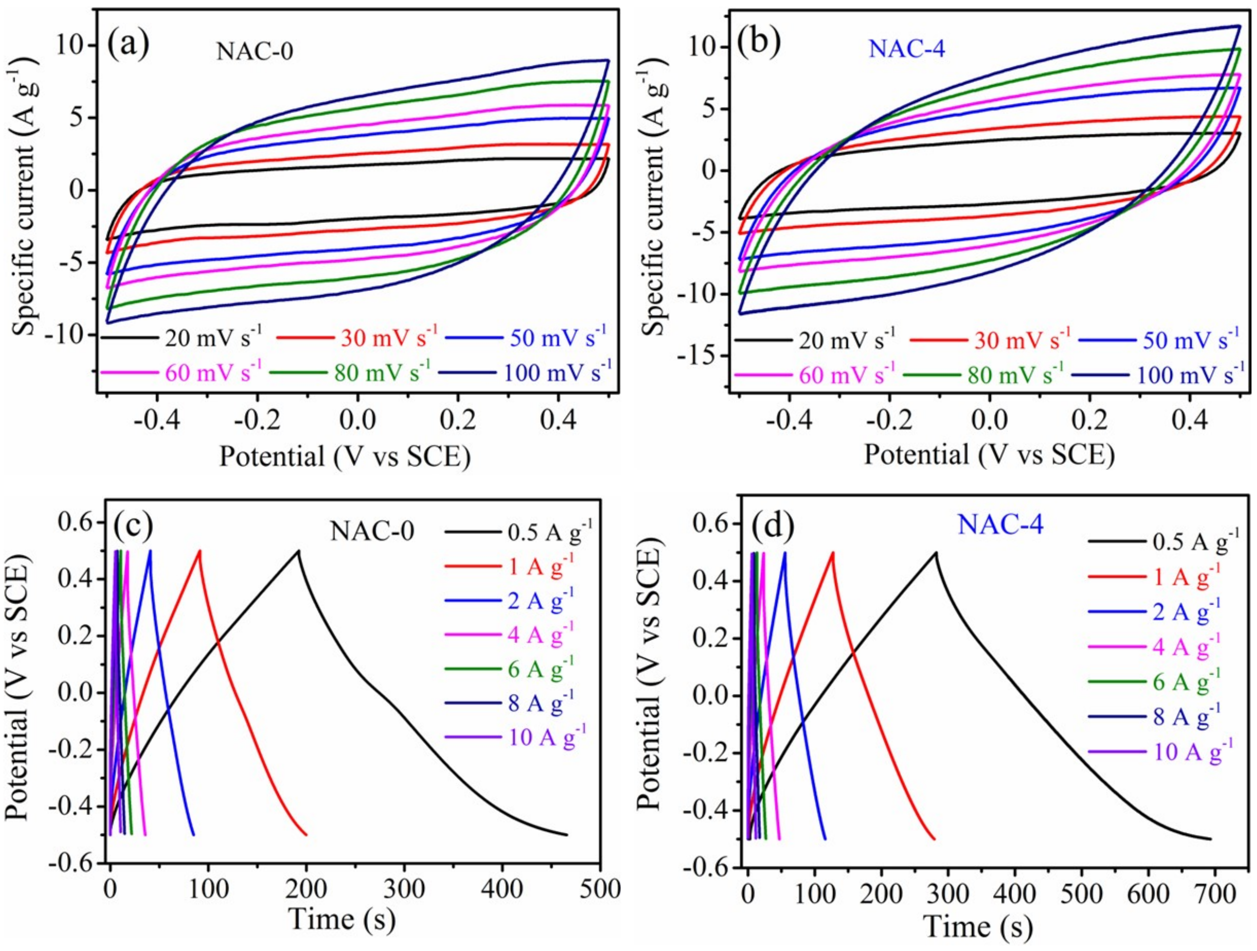

Fig. S4 CV profiles of (a) NAC-0 and (b) NAC-4 electrodes at different scan rates. GCD profiles of (c) NAC-0 and (d) NAC-4 electrodes at different current densities.

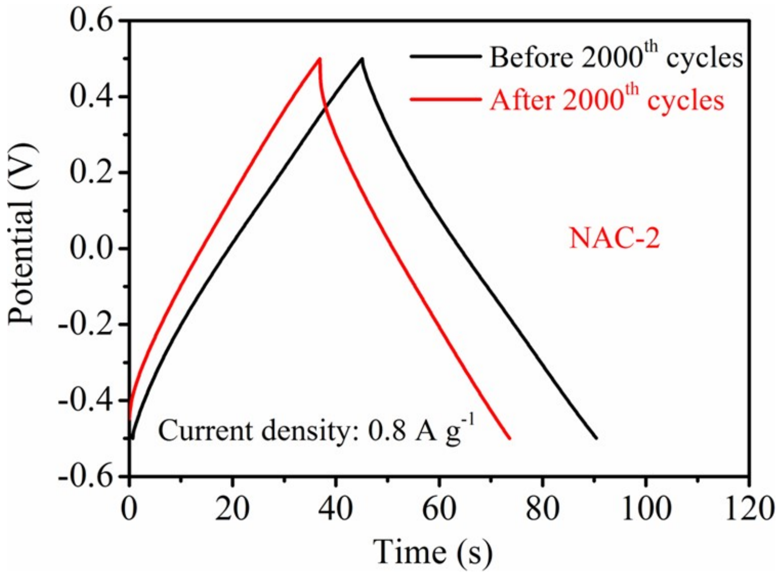

Fig. S5 Charge-discharge curves of NAC-2 electrode before and after 2000 galvanostatic charge-discharge (GCD) cycles at 0.8 A g^{-1}.

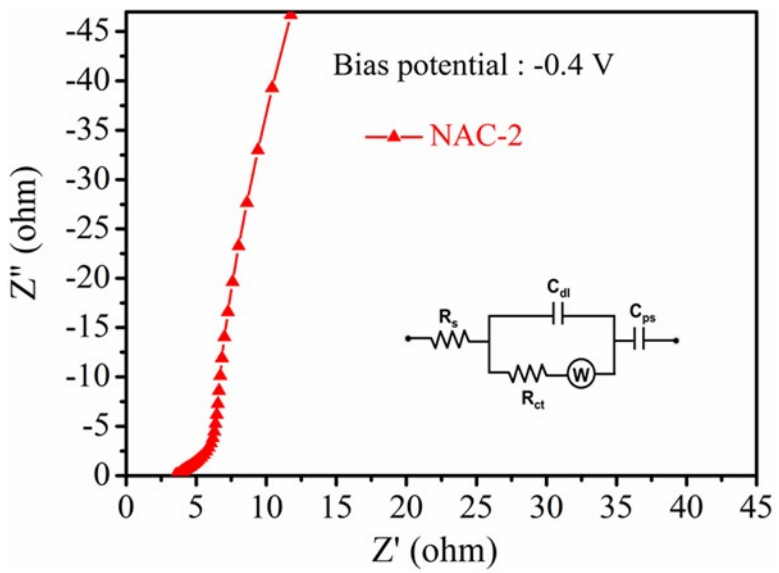

Fig. S6 Nyquist plot of NAC-2 electrode at -0.4 V and inset shows the equivalent circuit fit.

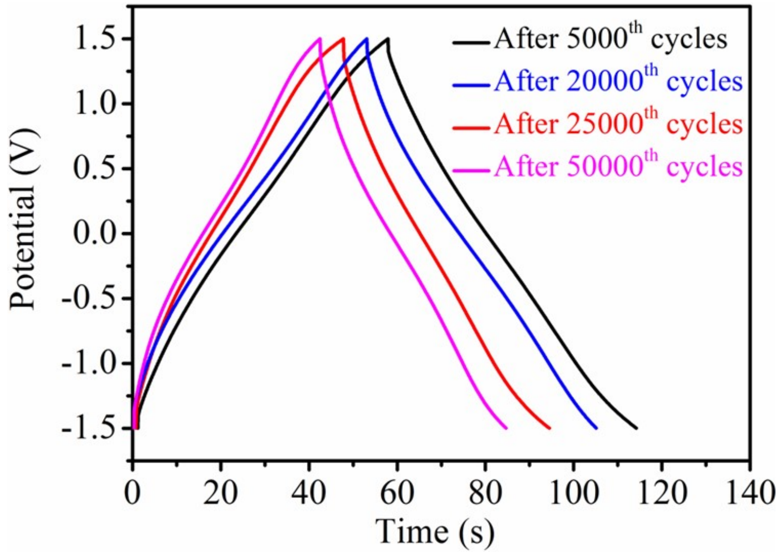

Fig. S7 Charge-discharge curves of the fabricated symmetric device at different intervals of cyclic tests.

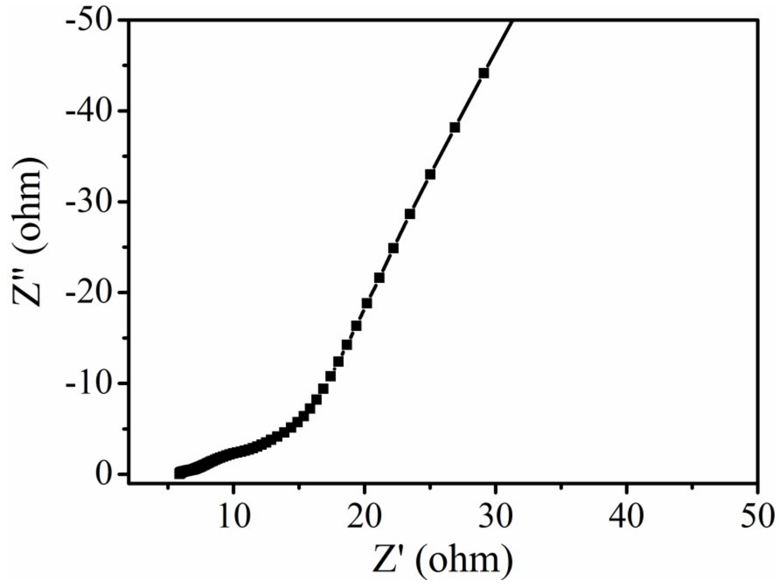

Fig. S8 Nyquist plot of NAC-2 electrode at -0.4 V in 0.1 M H_2SO_4/PVA solid electrolyte.
